# When Standing on a Moving Support, Cutaneous Inputs Provide Sufficient Information to Plan the Anticipatory Postural Adjustments for Gait Initiation

**DOI:** 10.1371/journal.pone.0055081

**Published:** 2013-02-04

**Authors:** Laurence Mouchnino, Jean Blouin

**Affiliations:** Cognitive Neurosciences Laboratory, Aix-Marseille University, CNRS, Marseille, France; McGill University, Canada

## Abstract

Gait initiation is preceded by initial postural adjustments whose goal is to set up the condition required for the execution of the focal stepping movement. For instance, the step is preceded by a shift of the body’s center of mass towards the stance foot unloading the stepping leg. This displacement is produced by exerting forces on the ground (i.e., thrust) while the body is still motionless. The purpose of this study was to identify whether the mere cutaneous inputs from the feet soles evoked by a lateral translation of the support could be used to scale the initial postural adjustments. Participants stood with their eyes closed on a force platform that could be moved laterally with a low acceleration (between 0.14 m/s^2^ and 0.30 m/s^2^) to reach a constant velocity of 0.02 m/s. This translation resulted in a change in the somatosensory cues from the feet soles without modifying vestibular inputs. Participants were instructed to produce a step with the right foot as soon as they felt the platform start to move (on either side) or heard an auditory cue. In the latter case, the platform stayed stationary. We found that the thrust duration was lengthened when the platform moved towards the supporting foot. In this condition, the cutaneous stimulation provided information related to a body shift towards the stepping leg. This increased thrust duration likely helped overcoming the non-functional body shift perceived towards the stepping leg. This result highlights the accuracy with which the actual standing position can be determined from foot sole cutaneous cues in the absence of visual and vestibular or proprioceptive inputs.

## Introduction

The safe and accurate execution of a step requires anticipatory postural adjustments (APAs) finely tuned to the context in which the movement occurs. In the case of gait initiation, these APAs are responsible for shifting the body weight both laterally towards the supporting side to unload the stepping leg, and forward to create the condition for forward progression [1]. MacKinnon et al. [2] found evidence that the APAs are progressively assembled and stored in advance of movement and triggered by corticospinal excitations. These authors showed that both the magnitude and duration of the APAs increased as the timing of a startle-like acoustic stimulus (which causes an early and automatic release of the planned movement) approached the expected “go” signal for step initiation. An important element in the scaling of the APAs before their dispatch to the periphery consists in determining the context of the step prior to its execution. The configuration of the body segments and the position of the body relative to the ground are examples of key elements that will have to be defined. This process is presumably based on sensory signals and might involve proprioceptive, cutaneous, visual and vestibular information [3], [4].

The scaling of the APAs is often performed without time pressure, as when initiating gait from a quiet standing position. In other circumstances, however, the APAs must be scaled promptly as when the body is subjected to an external perturbation. The possibility to process rapidly sensory information according to the changing context of the intended step was investigated by Timmann and Horak [5]. These authors asked subjects to produce a step forward as soon as they felt that the platform on which they were standing moved backwards. Although the stepping response occurred quickly after the platform translation (electromyographic responses had latencies of ∼200 ms), the APAs were well adapted to the changes in body state that resulted from the platform motion (see also [6]). While this study clearly showed the possibility to rapidly trigger off and shape the early APAs, it was not designed to provide information on the sensory cues responsible for detecting the change in the initial standing condition and for setting the APAs.

The initial phase of the APAs is referred to as the thrust. It corresponds to the vigorous lateral forces exerted on the ground, in the direction of the stepping side. These forces are responsible for accelerating the center of mass over the supporting leg and shifting the subject’s body weight. Ruget et al. [7] reported that the thrust of the APAs is unaffected when a change in leg muscle proprioception (through muscle vibration) occurs during its preparation (i.e., 400 ms before their initiation). This suggests that proprioception from the lower limb cannot be integrated rapidly to scale the thrust.

The fast and slow adapting cutaneous mechanoreceptors are highly sensitive to the forces applied to the sole of the foot and they provide reliable information about the direction and amplitude of the center of pressure motion (CoP) [8]–[12]. Reducing the transmission of the cutaneous input, for instance by tibial nerve block [11] or cooling the soles of the feet [13], [14] markedly perturbs gait (e.g., smaller propulsive force, change in weight distribution). Therefore, the cutaneous input appears as a good candidate for properly setting the forthcoming APAs.

The present study was designed to specifically test this possibility. More specifically, we asked participants to produce a complete step as soon as they felt the motion of the platform on which they were standing with their eyes closed (i.e., step-to-cue task). Importantly, because the platform acceleration was set below the vestibular threshold, it was deemed to be detectable through cutaneous plantar receptors. Considering the shear forces evoked by the platform motion, cutaneous receptors should provide information related to a leftward body displacement during rightward acceleration of the support surface (i.e., towards the stepping side). Inversely, leftward platform acceleration (i.e., towards the supporting side) should be sensed as a rightward displacement of the body. We reasoned that if the scaling of the thrust of the APAs is based on a rapid feedback loop involving foot cutaneous inputs, then smaller and greater thrusts should be observed during rightward and leftward platform accelerations, respectively.

## Materials and Methods

Eight participants aged from 22–28 years (mean age 25 years) without any known neurological and motor disorders, participated in this experiment. All participants gave their written informed consent to take part in this study, which conforms with the standards set in the Declaration of Helsinki. The local Ethics Committee (Sud Méditerranée 1, ID RCB : 2010–A00074-35) specifically approved this study. The participants stood at ease barefoot on a 60 × 120 cm force platform placed in the middle of a motor-driven treadmill. Their heels were 10 cm apart and their feet were at an angle of 30° from the anteroposterior axis regardless of their height. The participants’ frontal plane was in the mediolateral direction of the treadmill motion. The participants’ task was to produce with their eyes closed, a complete step with the right leg as soon as they either felt the platform moving (step-to-cue paradigms, [15]) or heard an auditory signal (a 50 ms beep, stationary platform condition) in order to land on a steady surface adjacent to the platform. The treadmill randomly translated either leftward or rightward or stayed stationary. The time elapse between the signal given to the participants to close their eyes and the treadmill motion or auditory signal was changed from trial to trial (i.e. between ∼2 s and ∼4 s) to minimize pre-planned movement initiation.

The participants performed 90 trials, 30 in each platform condition (i.e., translation towards the supporting side, translation towards the stepping side or stationary). The acceleration of the platform was set individually according to each subject’s motion detection threshold. This threshold was found prior to the experimental session and was defined as the lowest acceleration that produced motion detection in 5 consecutive trials, while participants stood in an erected position on the platform. During this set of trials, different ramp accelerations were used to bring the platform to a constant velocity of 0.02 m/s. The accelerations of the platform and their directions were pseudo-randomly selected from trials to trials. The motion detection threshold varied between 0.14 m/s^2^ and 0.3 m/s^2^ between participants (global mean = 0.21 m/s^2^±0.05). During the experimental session, the participants were accelerated according to their motion detection threshold until the platform moved at constant velocity. As it will be shown in the results section below, these low accelerations were too weak to evoke stretch reflexes.

To specifically control whether the vestibular system was also stimulated during the platform translations (together with the resulting cutaneous stimulation), we recorded for each participant, 12 additional trials during which the participants were asked to remain still before, during and after the translation. These trials were equally distributed in a random manner with the other experimental trials.

### Behavioural Recordings and Analyses

**Figure 1:**
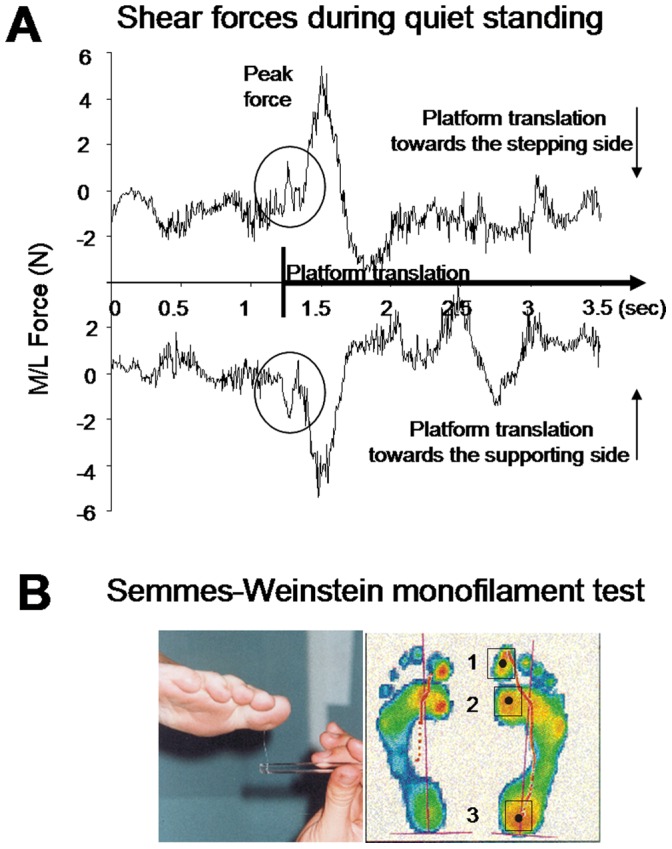
Translation-evoked shear forces and pressure detection test. ( A) representative lateral forces recorded during quiet standing for both platform translation sides. (B) illustration of the method used to identify the foot cutaneous threshold using the Semmes-Weinstein monofilament test and, of the three investigated foot areas.

The ground reaction forces and moments were recorded with an AMTI force platform (Advanced Mechanical Technology Inc., USA) at a sampling rate of 1000 Hz. The mediolateral forces (in N) were analysed because these shear forces underneath the feet were thought to stimulate the cutaneous receptors. The primary change of the force was synchronous with the platform translation onset and the direction of this force was opposite to that of the plateform motion (Fig. 1A). We calculated the peak of this force with respect to its baseline value (i.e., prior to translation onset). The displacements of the center of pressure (CoP) were also analyzed in the medio-lateral direction as changes in the thrust after platform motion were expected to occur in that direction. Their duration and amplitude were computed from the starting (i.e., neutral) position to the maximum CoP displacement. Medio-lateral head acceleration was measured by using an Entran accelerometer fixed on the participant’s head and digitally sampled at 1000 Hz, with a Keithley A/D converter device (AD-win pro, Keithley Instruments, Cleveland, OH). As head acceleration did not reach vestibular threshold for motion detection (i.e. 0.048 m/s^2^ [16]) before the peak medio-lateral force, head acceleration was calculated at the peak force-time.

The movement of the stepping leg was recorded by fixing a spherical reflexive marker on the malleolus lateralis and tracking its position with an automatic optoelectronic system (SMART, BTS Milano) at a frequency of 120 Hz. We calculated the foot excursion from the starting (neutral) position to the final forward foot position. The marker turned out to be defective for one subject. This subject was discarded from the kinematics analyses.

Bipolar surface electromyography (EMG, Bortec AMT- 8 system: Bortec Bomedical, Canada) was used to record the activity of 8 leg muscles (4 muscles on each side) that are known to be responsible for the lateral stability of the foot (i.e., tibialis posterior (TP) and fibularis longus (FL)) and for the anteroposterior stability of the ankle joint (i.e., tibialis anterior (TA) and gastrocnemius medialis (GM)). EMG signals were pre-amplified at the skin site and sampled at 500 Hz (band-pass filtered 20–250 Hz). To quantify EMG activity before and after the translation onset, we computed, for each trial, the integral of the full-wave rectified EMG (EMGi) during 100 ms time-windows, both pre- and post-platform accelerations. Then, we subtracted the EMGi post- from the EMGi pre-platform acceleration (i.e., ΔEMGi). Positive values of the computation would indicate increased EMG activity due to platform motion. In this case, the translation would not only stimulate cutaneous receptors but also muscular receptors (e.g. muscle spindles).

The Semmes-Weinstein monofilament test was used to provide data about skin sensitivity to mechanical stimuli of each foot. The test was carried out on the three areas of each sole that normally make contact with the ground during gait initiation (Fig. 1B): the big toe, the head of the first metatarsal innervated by the medial plantar nerve and the heel innervated by the medial calcaneal branch of tibial nerve. The test consisted of applying a series of a standardized set of hairs, all of constant length but varying in diameter, at a 90° angle against the skin, up until the point where it begins to bend. The force at which a monofilament bends is proportional to its diameter. The monofilaments were selected so as to give a linear scale of perceived intensity (i.e., a logarithmic scale of applied force). During the test, the participants were seated and relaxed with their eyes closed. The participants were simply asked to respond when they felt that their feet had been touched by the monofilaments.

Behavioural data were subjected to repeated measures analysis of variances (ANOVA) with condition (platform shift towards the supporting and the stepping sides, and stationary platform) as within-subject factor. Significant effects were further analysed using Newman-Keuls post-hoc tests. The level of significance was set at 5%. We also used the paired *t*-test and Wilcoxon non parametric test when specified.

## Results

### Sensory Stimulation Evoked by the Platform Translation

**Figure 2:**
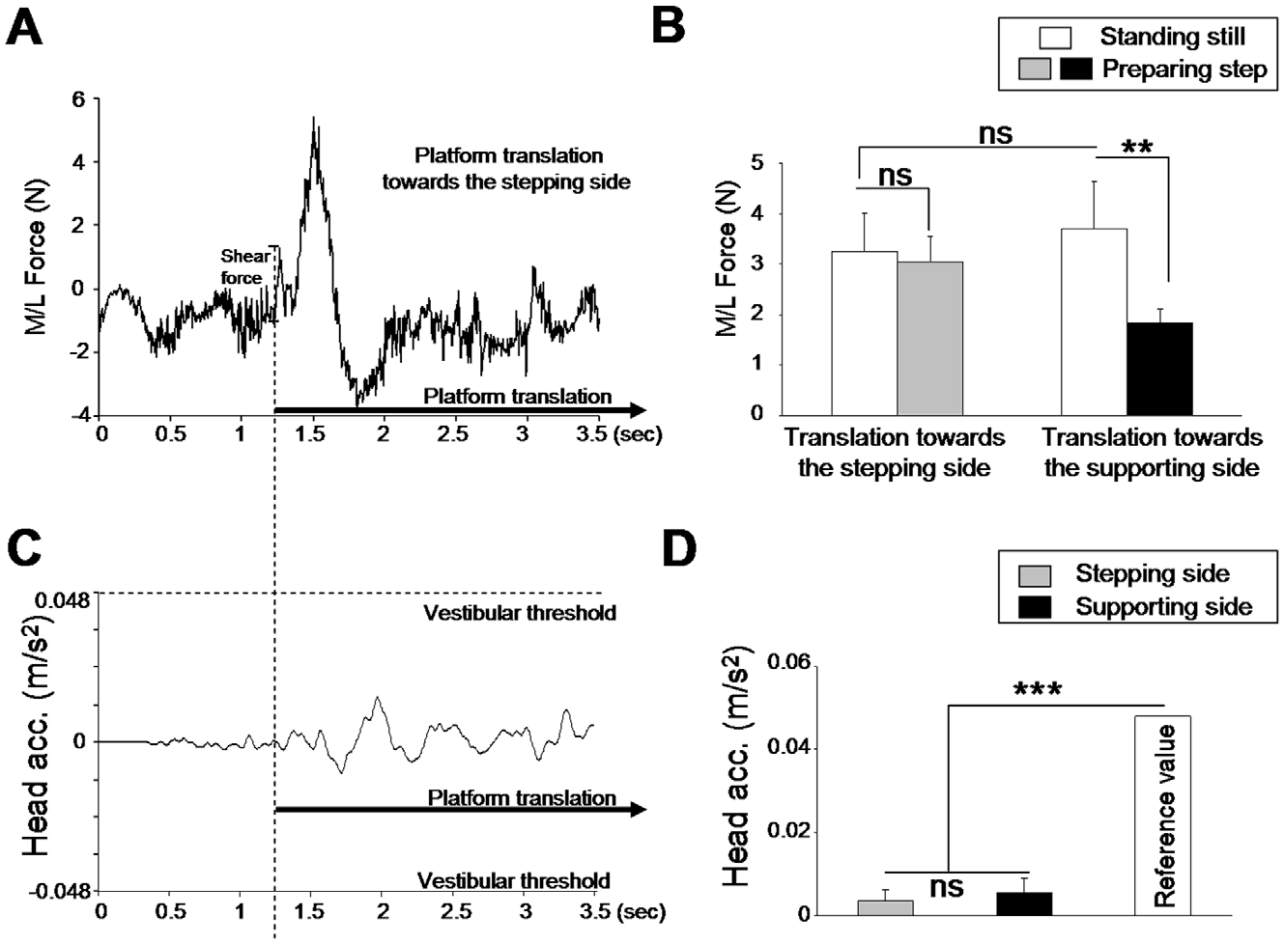
Shear forces and head acceleration. (A) lateral forces recorded during quiet standing position for a representative trial. The amplitude of the force was computed from the translation onset to the peak of the first shear force. (B) mean amplitude of lateral (shear) forces measured at the maximal peak force evoked by the platform translation when the participants were standing still or preparing to step forward. (C) head acceleration recorded during quiet standing position for the same represenative trial as above. (D) mean head accelerations measured at the time to peak force. ns: p>0.05, **p<0.01, ***p<0.001.

As shown in Figure 2A, a small but clear lateral shear force peaked early after the platform acceleration in the quiet standing task (ran before the main experimental session). All participants reported having sensed all platform movements. The direction of the shear force depended on the direction of the platform motion. This force, which presumably activated the sole receptors, resulted from the relative motion of the platform and of the feet. A subsequent and greater shear force was observed shortly after the first one (Fig. 1A). However this second shear force was absent when the participants had to produce the step. The initial shear force amplitude (on average 3.46N ±0.74) did not significantly differ between translation directions when the participants were asked to stand still (t_7_ = 1.49; p = 0.17, Fig. 2B).

More importantly, during the platform acceleration, the head acceleration (Fig. 2C) remained largely below the 0.048 m/s^2^ vestibular threshold reported by Gianna et al. [16] for acceleration-step stimuli. This was confirmed by the comparison of the head acceleration, recorded at the peak shear force, to a standard value set at the vestibular threshold of 0.048 m/s^2^ (Fig. 2D). Results of these analyses showed that head acceleration was significantly smaller than the vestibular threshold for both sides of platform translations (0.0035 m/s^2^±0.0025; t_7_ = −49.18; p<0.05 and 0.0054 m/s^2^±0.0035; t_7_ = −34.07; p<0.05 for translations towards the stepping and the supporting sides, respectively). Moreover, the ΔEMGi (i.e., difference -for each muscle- of EMGi pre- and post-platform accelerations) showed no effect of the direction of the support translation (rightward or leftward, F_1,7_ = 1.075; p = 0.33), no effect of muscles (TP, FL, TA, GM, F_3,21_ = 1.24; p = 0.31) and no side effect (right and left legs, F_1,7_ = 0.021; p = 0.88). Moreover, the ANOVA did not reveal any significant interaction between translation directions, muscles and sides. Therefore, the motion of the platform did not trigger direction-related muscle activation and/or inhibition or stretch reflex responses. Hence, analyses of head acceleration and muscle activity suggest that the perception of the small platform accelerations used in the present study arose from cutaneous receptors rather than from vestibular receptors or muscle proprioception.

**Figure 3:**
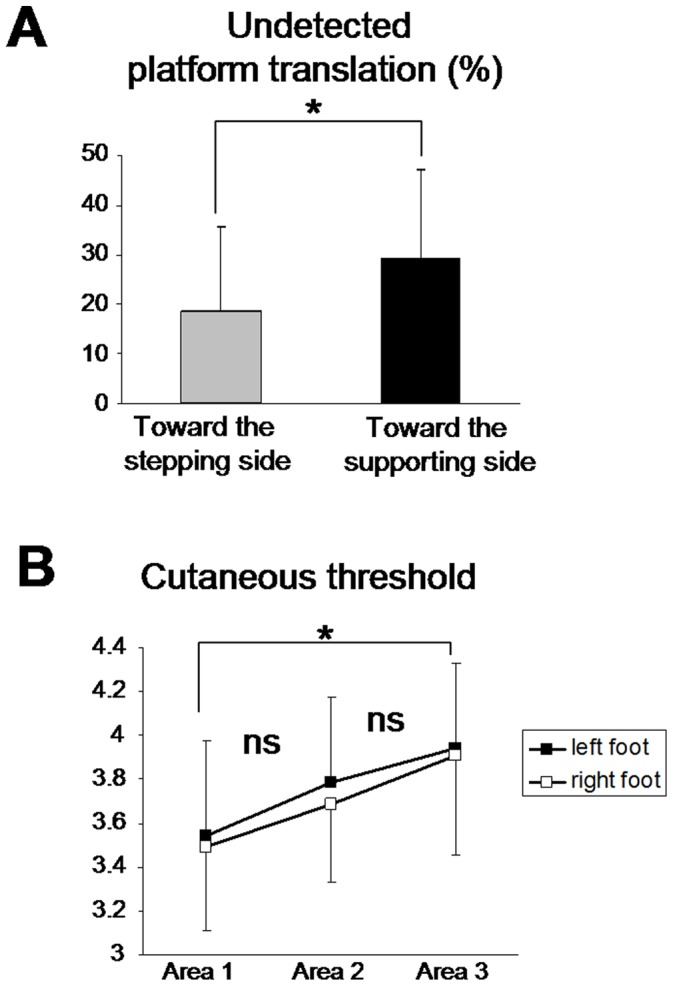
Undetected platform translation and cutaneous threshold. ( A) Mean percentage of undetected translations of all subjects. (B) mean cutaneous threshold of the three areas of the foot sole surface in contact with the ground during gait initiation. ns: p>0.05, *p<0.05.

Unexpectedly, in several trials of the main experimental session (i.e., step-to-cue task), the participants did not perceive the translation of the platform. Using the Wilcoxon non parametric test, we found that participants detected the translations significantly better when they were directed towards the stepping side (19% of undetected trials) than towards the supporting side (i.e., 29%, z = 2.36; p = 0.017) (Fig. 3A). Trials with undetected platform translations were repeated during the experimental session. The difference of undetected percentage of trials between both motion directions might result from a difference in the cutaneous sensitivity between the two feet (Fig. 3B). Therefore we submitted the cutaneous perceptual threshold (as measured with the Semmes-Weinstein monofilament test) to a 2 feet (right and left) x 3 areas (big toe, head of the first metatarsal, and heel) ANOVA. The ANOVA did not reveal a significant effect of foot (F_1,7_ = 0.68; p = 0.43) indicating that the difference of the number of undetected trials cannot be explained by different thresholds for detecting pressure between the feet. A significant main effect of foot area was observed (F_2,14_ = 4.07; p = 0.04); the threshold being higher for the heel than for the big toe (Fig. 3B).

Surprisingly, we found that the mere preparation to produce a step modified the amplitude of the passive shear forces evoked by the translation (Fig. 2B). This was confirmed by the significant interaction between task and side (F_1,7_ = 13.65; p = 0.007). The decomposition of the interaction showed that the shear forces evoked by the translation towards the supporting side were smaller (1.8 N ±0.2, Fig. 2B) when the participants were preparing to produce a step than when they simply had to remain still on the platform (3.7 N ±0.9). The forces produced in the former condition were also significantly smaller than in both tasks with translations towards the stepping side. The decrease of the shear forces evoked by the platform translation in the stepping task could have contributed to the increased number of undetected trials that was observed in that condition.

### Effects of Platform Translation on the APAs

**Figure 4:**
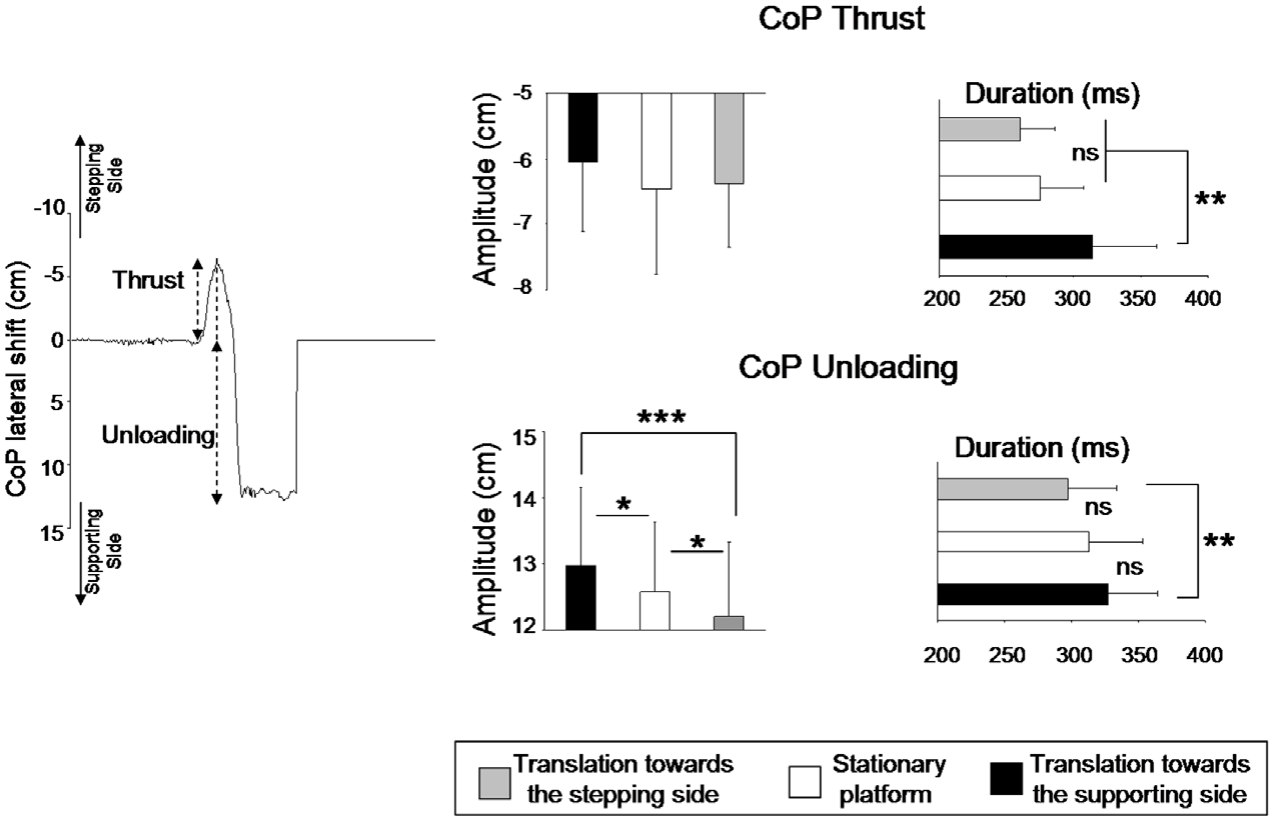
CoP lateral displacement. Lateral CoP recorded during stepping for a representative trial (left panel). Mean amplitude and duration of both the thrust and the subsequent unloading component of the APAs (right panel). ns: p>0.05, *p<0.05, **p<0.01, ***p<0.001.

On average, participants initiated the thrust 329 ms ±56 after the onset of the platform translation. A t-test analysis revealed that the thrust latencies did not differ between translation sides (i.e., towards the stepping side and towards de supporting side) (t_7_ = 0.48; p = 0.63). To determine whether the setting of the APAs (i.e., CoP thrust and unloading, Fig. 4) depended on the platform translation, we submitted the amplitude and duration of both the thrust and unloading component to separate one-way ANOVAs with three levels (translation towards the stepping and supporting sides and stationary). The platform conditions did not have a significant effect on the thrust maximal amplitude (overall mean −6.3 cm ±1.06; F_2,14_ = 2.41; p = 0.12) but they significantly affected the thrust duration (F_2,14_ = 12.99; p = 0.0006) (Fig. 4). The thrust duration was longer (314 ms ±47) when the platform translated towards the supporting side than when the platform either translated towards the stepping leg (260 ms ±25) or stayed stationary (275 ms ±32). Increasing the thrust duration when the platform moved towards the supporting side likely helped to overcome the perceived non-functional body shift towards the stepping leg.

To verify whether the body was moving before the thrust initiation, we compared the latencies of the head acceleration and of the thrust onset. Separate t-test analyses revealed that both latencies did not significantly differ for both translation sides (both p>0.05). This result provides an additional indication that the thrust, which occurred at a similar time as the head acceleration, could not be planned on vestibular-based information evoked by platform acceleration.

**Figure 5:**
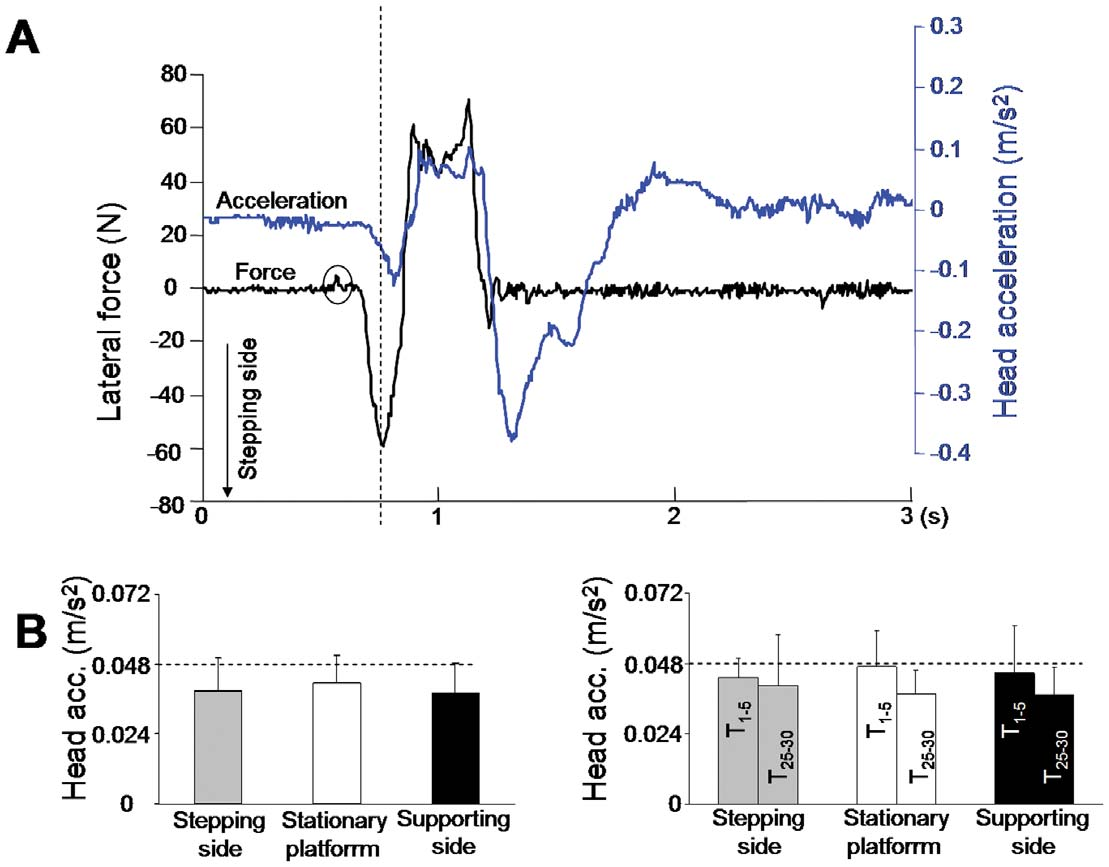
Ground lateral forces and head acceleration during stepping movement. ( A) representative lateral force exerted onto the ground (left scale) and associated head acceleration (right scale) recorded when a participant produced a step. The shear force evoked by the platform motion towards the stepping side is indicated by a circle. (B) mean head acceleration recorded at the time of the peak thrust (left panel) and mean head acceleration computed for the first (T_1–5_) and the last (T_25–30_) 5 trials (right panel). The vestibular threshold was indicated by the broken lines.

After the CoP thrust, and as shown in Figure 4, the body weight was transferred towards the supporting foot. This unloading phase of the APAs allowed participants to free their stepping leg and initiate the step. The motion of the standing platform greatly affected this phase, as revealed by the significant effects of the platform conditions on both the amplitude (F_2,14_ = 13; p = 0.001) and duration (F_2,14_ = 6.64; p = 0.009) of the unloading component. Compared to its amplitude in the stationary platform condition, the unloading was respectively greater and smaller than when the platform moved towards the supporting side (13 cm ±1.2) and moved towards the stepping side (12.20 cm ±1.12). The ANOVAs and the post-hoc test showed that when the platform was shifted towards the supporting side, the unloading was significantly longer (327 ms ±36) than when the platform moved towards the stepping side (297 ms ±35). Together, these results showed that when the translation-evoked body shift was opposed to that planned by the motor command, both the thrust and the unloading increased to enable the stepping movement.Typical examples of lateral force and head acceleration time-series signals are shown in Figure 5A. The figure shows that head acceleration slightly increased while the forces were exerted onto the ground during the thrust. Comparisons with the standard 0.048 m/s^2^ (i.e. vestibular threshold, [12]) revealed that at the time of the peak force, head acceleration (Fig. 5B) was not significantly different to the acceleration required to stimulate the vestibular system in both the stationary platform condition (0.041 m/s^2^±0.009, t_7_ = −1.89, p = 0.09) and in the stepping side platform condition (0.038 m/s^2^±0.011, t_7_ = −2.27, p = 0.056). On the other hand, when the platform translated towards the supporting side, the head acceleration was still below the threshold value (0.037 m/s^2^±0.01; t_7_ = −2.69; p = 0.03). Overall these results suggest that the acceleration of the head was too weak and occurred too late to have a significant contribution in both the planning and execution of the CoP thrust.

In the context of a possible translation of the platform, the participants may have adopted an adaptive strategy consisting in increasing the stiffness of their body to minimize body motion (and therefore head acceleration ). To test for the use of such strategy, we compared for each subject the mean head acceleration recorded at the peak thrust for the 5 first and 5 last trials (T_1–5_ and T_25–30_, respectively). We submitted the mean head accelerations to a 3 conditions (stationary platform, rightward and leftward platform accelerations)×2 trials sets (T_1–5_; T_25–30_) repeated-measures ANOVAs. The ANOVA did not reveal a significant effect of condition (F_2,14_ = 0.2; p = 0.81) suggesting that the randomized order of the trials prevented any specific adaptation to the platform translation. A significant main effect of trials sets was however observed (F_1,7_ = 15.95; p = 0.005), the head acceleration being greater in the first 5 trials (T_1–5_ = 0.045±0.0018) than in the 5 last trials (T_25–30_ = 0.038±0.0035) (Fig. 5). This suggests that the participants increased their body stiffness across the experimental session regardless of the conditions (i.e., stationary or moving platform).

In order to compare the stepping movements in the different conditions, we analyzed the duration and amplitude of the forward foot excursion. The duration of the stepping movement was not significantly modified by the conditions (on average 690 ms ±51; F_2,12_ = 2.22; p = 0.15). The forward excursion of the stepping foot was slightly, but significantly greater in the stationary condition (61.4 cm ±2.6, F_2,12_ = 12.01; p = 0.001) than in the two translation conditions which did not show significant difference (overall mean, 60 cm ±2.6, p = 0.59). The shorter step excursion computed in the sagittal plane when the support moved on either side, was likely the result of the deviation of the whole body from that plane in the conditions with platform displacements.

## Discussion

The main finding of the present study was that participants were able to use cutaneous inputs evoked by the motion of the support translation, to scale the APAs accordingly. Indeed, the mere transient changes in the contact forces (shear forces) between the feet and the platform were able to trigger consistent changes in the forthcoming APAs. These changes could be observed at the early stage of the APAs (i.e. the thrust), that is ∼330 ms after the platform translation. These findings corroborate those reported by Timman and Horak [5] showing that the APAs can be set rapidly according to the direction of the base of support motion. However, this previous study was not designed to determine the sensory signals that allowed the quick setting of the APAs (for instance, the participants had their eyes open and their head acceleration was not recorded). Several key factors of the present study argue for a prevailing contribution of cutaneous inputs in the APAs regulation. For instance, the changes in the APAs occurred despite the fact that visual information was unavailable (i.e., participants had their eyes closed) and that head acceleration remained under the detection threshold of the vestibular system during platform translations. As such, this result supports Fitzpatrick and McCloskey’s [17] suggestion that large disturbances of posture is required before vestibular mechanisms can provide perceptual information about body sway. In addition, Honeycutt et al. [18] showed in the decerebrated cat, that robust and directionally appropriate muscle activation and/or inhibition was present without the cerebral cortices. Their observations on the muscles acting on the hip, knee and ankle joints of the hind limb strongly argued that the spinal cord and brain stem are responsible for the directional information driving appropriate muscular responses to horizontal perturbations. However in our study, we found no evidence of platform motion-induced muscular activation (e.g. directionaly based response or stretch-reflexes), suggesting little or no contribution of proprioceptive information. These important elements thus provide compelling evidence that the changes in the APAs observed during the platform translation largely relied on cutaneous cues from the plantar sole rather than from vestibular or proprioceptive information. Despite that foot intrinsic muscles (not recorded) have been found to have little contribution during double leg stance compared to single leg stance [19], further experiments are necessary to determine their specific role in the setting of the APAs.

Our results extend those reported by Bent et al [20] that did not show APAs modifications after vestibular stimulation and by Inglis and Macpherson [21] in cats, that demonstrated that the pattern of force response after a translation of the support was unaffected by bilateral vestibular loss. Besides, the role of the cutaneous afferents in the fine control of the mediolateral shear forces has also been reported in cats during locomotion. Indeed Bouyer and Rossignol [22] showed that after cutting selectively cutaneous nerves at the ankle level of cats, there was a large increase (>200%) in the mediolateral force (i.e., shear force) while vertical forces were very similar to the intact cats. That huge increase of the lateral force during overground locomotion after denervation and the fine setting of the APAs in the current study suggest that cutaneous inputs are well suited to control mediolateral balance during gait initiation as well as during locomotion.

Interestingly, the results of the present study markedly differed from those obtained by Ruget et al. [7] who tested the contribution of proprioception in the setting of the APAs prior to a step. In that study, a change in proprioceptive inputs was introduced by 1 second high frequency (80 Hz) muscle tendon vibrations at the ankle joints that started 400 ms before the thrust onset. Bilaterally placed over the ankles, the vibrators evoked proprioceptive-afferent inflow related to body tilt, towards either the supporting or stepping sides (i.e., similar to the lateral motion produced here). The change in the afferent inflow had no effect on the thrust, suggesting little contribution of proprioception in the rapid setting of the APAs. However, the thrust duration was significantly reduced when the ankles were vibrated at a 40 Hz frequency to activate the cutaneous afferents (essentially the Meissner corpuscles, [23]). As the cutaneous stimulation provided poor spatial information in Ruget et al.’s study (i.e., it did not evoke a well defined oriented body movement), it proved difficult to ascertain if cutaneous receptors, especially those from the plantar sole, could be processed to finely and rapidly tuned the thrust of the APAs. For instance, the change in the thrust observed after stimulation of the skin may have simply resulted from a protective strategy by the CNS to decrease the whole body acceleration towards the supporting leg following unexpected cutaneous stimulation of the ankle area.

The fact that we observed modifications of the thrust for only one direction of platform translation suggests that they resulted from directional-based online control during the APAs preparation, rather than from pre-set modifications induced by a cutaneous-evoked startle like signal. The thrust duration was lengthened when the platform translated towards the supporting leg side. In this condition, the cutaneous cues provided information relative to an undesired body shift towards the stepping leg. Such body motion was opposed to the planned body shift towards the supporting leg and could have prevented the required unloading of the stepping leg to move the foot. Therefore, in this condition, the CNS may have sought to increase the thrust duration to create the additional force necessary to overcome the effect of the passive body shift towards the stepping leg. Hence, the increase of both the amplitude and duration of the following unloading component of the APA that we observed in this condition may have been a direct consequence of the change in the thrust.

The mild assistance for shifting the body towards the supporting leg did not elicit changes in the APAs. This finding is in accord with those reported earlier showing that the CNS is more prompted to update the central feedforward command of the APAs for perturbations that shift the body towards the stepping leg than for those that move the body towards the supporting leg [24]–[26].

Also worthy of note is the fact that participants did not perceive ∼25% of the platform translations in the present step-to-cue task. This is in large contrast to what we observed in the session ran before the experimental task where participants did not have to produce a step after detecting platform motion. In this session, all platform motions were detected despite that their acceleration and velocity were similar to those employed in the step-to-cue task. The greater inability to detect the tactile sensory cues in the step-to-cue task could be related to the known somatosensory gating phenomenon that precedes and accompanies voluntary movements [27]–[30]. For instance, Shimazu et al. [27] found that the cortical response evoked by a somatosensory stimulation is attenuated when the stimulation is used as an imperative signal for triggering motor actions (compared to when participants do not have to react to it). In the present study, similar sensory gating may have affected the detection of the platform motion when participants had to trigger their stepping movements after having sensed the displacement.

Participants’ perception of platform motions was however strongly dependent on the direction of the surface displacements. Those towards the stepping leg side were indeed better detected than those towards the supporting side. Although the reason for the effect of motion direction cannot be formally elucidated here, it may be related to the expected shear forces produced during the thrust. This idea is partly based on Tokuno et al.’s [31] findings showing that participants have greater postural responses when the position of their CoP is opposite to the direction of the surface translation. In the present study, moving the platform towards the stepping side (passively) moved the CoP in the direction opposite to the forthcoming CoP thrust (actively) produced by the participants. Speculating on possible extension of Tokuno et al.’s [31] results, one can imagine that the preparatory processes related to the forthcoming stepping movement and APAs (e.g. those related to the expected cutaneous stimulation, see [32]) could have been engaged before the platform motion and favored detection of incongruent surface displacement in the opposite direction to the expected shear force during the thrust.

The question related to the changes in the forces exerted onto the ground to initiate the body weight transfer towards the supporting side becomes important for instance when it applies to ageing. For instance, incorrect weight shifting has been attributed as the leading cause of falls among the elderly (i.e. 41% of falls [33]). In that respect, two reasons emphasize the need of a sensory (i.e., cutaneous) rehabilitation in the elderly people. First of all, our observations make the cutaneous inputs and more particularly the fast adapting type that outnumbered the slow adapting in the foot sole [34], well suited to rapidly scale the APAs that initiate the body’s weight shift towards the supporting side. Second of all, the current body of knowledge in elderly people [see [35] for a review] indicates elevated thresholds in the foot that result from both a reduced number of receptors and an increased skin thickness.
